# “How to measure the outcome in the surgical treatment of vertebral compression fractures? A systematic literature review of highly cited level-I studies”

**DOI:** 10.1186/s12891-021-04305-6

**Published:** 2021-06-24

**Authors:** Sonja Häckel, Angela A. Renggli, Christoph E. Albers, Lorin M. Benneker, Moritz C. Deml, Sebastian F. Bigdon, Sufian S. Ahmad, Sven Hoppe

**Affiliations:** 1 Department of Orthopaedic Surgery and Traumatology, Inselspital, Bern University Hospital, University of Bern, Freiburgstrasse 18, 3010 Bern, Switzerland; 2 Centrum für Muskuloskeletale Chirurgie (CMSC), Charité – Universitätsmedizin Berlin, Augustenburger Platz 1, 13353 Berlin, Germany

**Keywords:** Osteoporosis, Spine, Vertebral compression fracture, Outcome measure, Citation density

## Abstract

**Background:**

The economic burden of vertebral compression fractures (VCF) caused by osteoporosis was estimated at 37 billion euros in the European Union in 2010. In addition, the incidence is expected to increase by 25% in 2025. The recommendations for the therapy of VCFs (conservative treatment versus cement augmentation procedures) are controversial, what could be partly explained by the lack of standardized outcomes for measuring the success of both treatments. Consensus on outcome parameters may improve the relevance of a study and for further comparisons in meta-analyses. The aim of this study was to analyze outcome measures from frequently cited randomized controlled trials (RCTs) about VCF treatments in order to provide guidance for future studies.

**Material and methods:**

We carried out a systematic search of all implemented databases from 1973 to 2019 using the Web of Science database. The terms “spine” and “random” were used for the search. We included: Level I RCTs, conservative treatment or cement augmentation of osteoporotic vertebral fractures, cited ≥50 times. The outcome parameters of each study were extracted and sorted according to the frequency of use.

**Results:**

Nine studies met the inclusion criteria. In total, 23 different outcome parameters were used in the nine analyzed studies. Overall, the five most frequently used outcome parameters (≥ 4 times used) were the visual analogue scale (VAS) for pain (*n* = 9), European Quality of Life–5 Dimensions (EQ-5D; *n* = 4) and Roland–Morris Disability Questionnaire (RMDQ, *n* = 4).

**Conclusion:**

With our study, we demonstrated that a large inconsistency exists between outcome measures in highly cited Level I studies of VCF treatment. Pain (VAS), followed by HrQoL (EQ-5D) and disability and function (RMDQ), opioid use, and radiological outcome (kyphotic angle, VBH, and new VCFs) were the most commonly used outcome parameters.

**Supplementary Information:**

The online version contains supplementary material available at 10.1186/s12891-021-04305-6.

## Introduction

Osteoporosis is a systemic skeletal disease that is characterized by a loss of bone density and microarchitecture and a resulting increase in bone fragility and thus the susceptibility to fractures [1]. The most common osteoporotic fractures are non-traumatic hip fractures followed by vertebral compression fractures (VCF) and forearm fractures [2]. In postmenopausal women, in particular, the incidence of osteoporotic VCF increases with age. For example, the lifelong risk of a 50-year-old Caucasian woman suffering from VCF is 16% [3, 4]. The consequence of this can be the loss of daily activities [5] and an up to an eight-fold increase in mortality [6]. In addition, the decrease in disability-adjusted life years (DALY) due to osteoporotic VCFs even exceeds that of common cancers [5].

Treatment of patients with osteoporotic VCFs is either conservative or surgical. Conservative treatment consists of pain relievers, early mobilization and radiological follow-up examinations to check the stability of the fracture. In contrast, surgical treatment mainly involves cement augmentation procedures of the fractured vertebrae [7]. Technically, a distinction is made between vertebroplasty (VP) and kyphoplasty (KP) [8]. While both are minimally invasive and percutaneous procedures, the difference, however, is that with KP the cement is applied into a cavity of the vertebral body (VB) previously created by a balloon. In contrast, the VP does not take this step. These two treatment strategies aim to achieve short-, medium- and long-term pain control as well as a reduction in disability, morbidity and mortality. This must be accompanied by antiosteoporotic drug therapy, as this is the basic intervention for patients with osteoporotic fractures [9]. In addition, if the VB height is not restored, spinal alignment may change, which is also related to other comorbidities, such as the risk of subsequent pulmonary death [10].

Despite a large number of published studies, there is no consensus on whether cement augmentation procedures for VCFs are advantageous in terms of achieving the predefined treatment goals compared to non-surgical treatment [7, 11]. This is noteworthy considering the fact that this treatment has been done frequently over the past 20 years. Although a majority of the published studies advocate cement enlargement, many of these studies use a retrospective study design and show no statistical significance [11]. Furthermore, there is a large variation in outcome parameters, which affects comparability between these studies. These outcome parameters include Health-Related Quality of Life (HRQoL), disability and function, and the radiological result.

The increasing number of osteoporotic VCF requires guidelines in clinical decision-making. To plan a Level I clinical trial, selecting the appropriate outcome measures is a challenging task. However, it is essential to carefully select these parameters to demonstrate adequate effects in clinical trials [12]. This systematic review aims to extract the outcome parameters from the most cited studies on VCF treatment to guide future study designs and clinical decisions.

## Materials and methods

We carried out a systematic review by following the PRISMA declaration (Preferred Reporting Items for Systematic Reviews and Meta-Analysis) [13]. As this study is based on public literature, it does not apply to ethical approval.

**Fig. 1:**
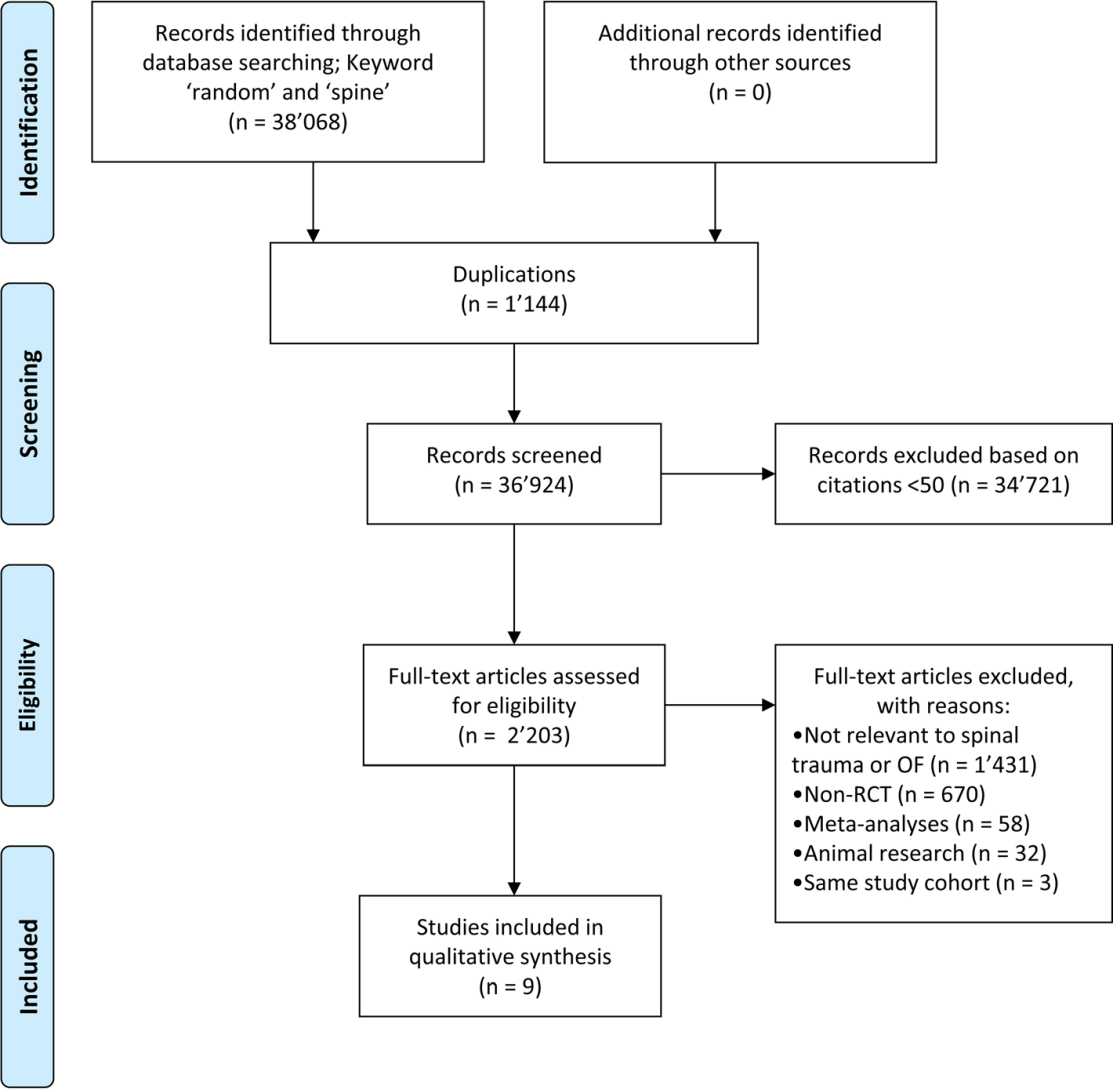
Selection process of studies. OF = Osteoporotic fracture; RCT = Randomized controlled trial

All articles on osteoporotic spinal fractures between 1973 and 2019 were identified in each journal (medical and non-medical) using the Web of Science Core Collection. Inclusion criteria were (1) treatment of osteoporotic vertebral body fractures in humans with cement augmentation (VP or KP); (2) Level I randomized controlled trials (RCT) based on the definition of the “Oxford Center for Evidence-Based Medicine (CEBM)” [14]; (3) more than 50 citations. Exclusion criteria were (1) animal studies, spondyloarthritis, medical therapy, exercise therapy, and traumatic osteoporosis; (2) non-clinical studies, systematic reviews and meta-analyzes; (3) fewer than 50 citations. A multi-step approach was used to identify level 1 studies [14] with ≥50 total citations addressing VCF (Fig. 1). Five hundred and twenty-four articles met the inclusion criteria. A further 513 papers were excluded based on the exclusion criteria after studying the abstract.

## Results

Nine studies, all published between 2009 and 2011, met the inclusion criteria for this review (Fig. 1).

Of the included studies, three were published in general medical journals (The New England Journal of Medicine, Lancet), three in spine research related journals (Spine, Journal of Neurosurgery-Spine), one in a radiological journal (the American Journal of Neuroradiology), and two in osteoporosis/ bone research journals (Osteoporosis International, Journal of Bone and Mineral Research).

Five studies came from Europe, two from Asia, one from the USA, and one from Australia. The total number of citations was between 60 to 561 with a citation density of 9 to 70 per year. One study was declared an industry-sponsored trial [15]. The two studies with the highest number of citations were published by Kallmes et al. [16] and Buchbinder et al. [17] both in the New England Journal of Medicine (NEJM) (Table 1).

**Table 1:** Summary of overall citation and citation density of included studies

Rate	Study	Journal	Year	Interventions performed	Total citations	Citation density (Citations/ year)
1.	Kallmes et al. [16]	NEJM	2009	VP vs Sham	561	70
2.	Buchbinder et al. [17]	NEJM	2009	VP vs Sham	554	69
3.	Klazen et al. [18, 19]	Lancet	2010	VP vs conservative	299	43
4.	Rousing et al. [20]	Spine	2010	VP vs conservative	104	15
5.	Liu et al. [21]	Osteoporosis International	2010	VP vs KP	102	15
6.	Farrokhi et al. [22]	Journal Neurosurgery Spine	2011	VP vs conservative	72	12
7.	Boonen et al. [15]	JBMR	2011	KP vs conservative	65	11
8.	Blattert et al. [23]	Spine	2009	KP (CaP vs PMMA)	62	8
9.	Klazen et al. [19]	American Journal of Neuroradiology	2010	VP vs conservative	60	9

NEJM = New England Journal of Medicine, JBMR = Journal of Bone and Mineral Research, VP = Vertebroplasty, KP = Kyphoplasty, CaP = Calcium phosphate, PMMA = polymethylmethacrylate.

### Interventions performed

The following objectives were analysed in the included studies: VP/KP versus conservative treatment (*n* = 5), VP versus sham procedures (*n* = 2), VP versus KP (*n* = 1), and different cement formulations for KP (n = 1) (Table 1).

### Outcome parameter

The absolute use of all outcome parameters was analyzed regarding their type (pain, HRQol, function and disability, radiographic imaging and others). In total, 23 different outcome parameters were used in the nine analyzed studies. Ten different outcome parameters were used to analyze the HRQol, five different parameters for radiographic imaging, four for disability and function and one for pain (Table 2, Table S1 of supplemental material). Overall the five top used outcome parameters (≥ 4 times used) were: Visual analogue scale (VAS-pain; *n* = 9), European Quality of Life–5 Dimensions (EQ-5D Score; *n* = 4) and Roland–Morris Disability Questionnaire (RMDQ; n = 4) (Table 2).

**Table 2:** Publications included in the analysis according to the inclusion criteria

Study	Pain	Health-related Quality of Life	Disability/Function	Radiographic	Other	Primary outcome parameter
Kallmes et al. [16]	VAS	EQ–5D SF-36 (PCS, MCS), SOF–ADL score, CMI, Pain Frequency and Pain Bothersomeness Indices	RMDQ	_	Opioid use	RMDQ at 1 month
Buchbinder et al. [17]	VAS (Overall pain* and pain at rest and pain in bed at night)	EQ–5D QUALEFFO AQoL	RMDQ	_	_	Overall pain at 3 months
Klazen et al. [18, 19]	VAS	EQ-5D QUALEFFO	RMDQ	_	cost-eff effectiveness	Pain at 1 month and 1 year
Rousing et al. [20]	VAS	SF-36 DPQ	Timed Up&Go, MMSE, Barthel, Chair Test	plain radiographs (fracture detection)	_	Not defined
Liu et al. [21]	VAS	_	_	VBH, kyphotic wedge angle	_	Not defined
Farrokhi et al. [22]	VAS	ODI	_	VBH and SI, new fractures	_	Pain and ODI
Boonen et al. [15]	VAS	SF-36 (PCF) EQ-5D	RMDQ	_	patient satisfaction	SF-36 (PCF) at 1 month
Blattert et al. [23]	VAS	_	_	bisegmental endplate angle on plain radiographs; distribution and texture of cement plugs on 2-mm CT-scans	_	Not defined
Klazen et al. [19]	VAS	_	_	New VCFs	_	New VCFs

Citation density (Citations/ year); AQoL = The Assessment of Quality of Life; cons. = conservative treatment; JBMR = Journal of Bone and Mineral Research; KA = Kyphotic angle; KP = kyphoplasty; MRI = Magnetic Resonance Imaging; NEJM = The New England Journal of Medicine; QUALEFFO = Questionnaire of the European Foundation for Osteoporosis; VCF = Vertebral compression fracture; VP = Vertebroplastie; DPQ = Dallas Pain Questionnaire; EQ-5D = European Quality of Life–5 Dimensions; MCS = Mental component score;mmSE = Mini-Mental State Examination; ODI = Oswestry Disability Index; PCS = Physical component score; RMDQ = Roland–Morris Disability Questionnaire; SF 36 = Short Form 36 General Health Survey; SI = Sagittal index; VBH = Vertebral body height.

### Pain

**Fig. 2:**
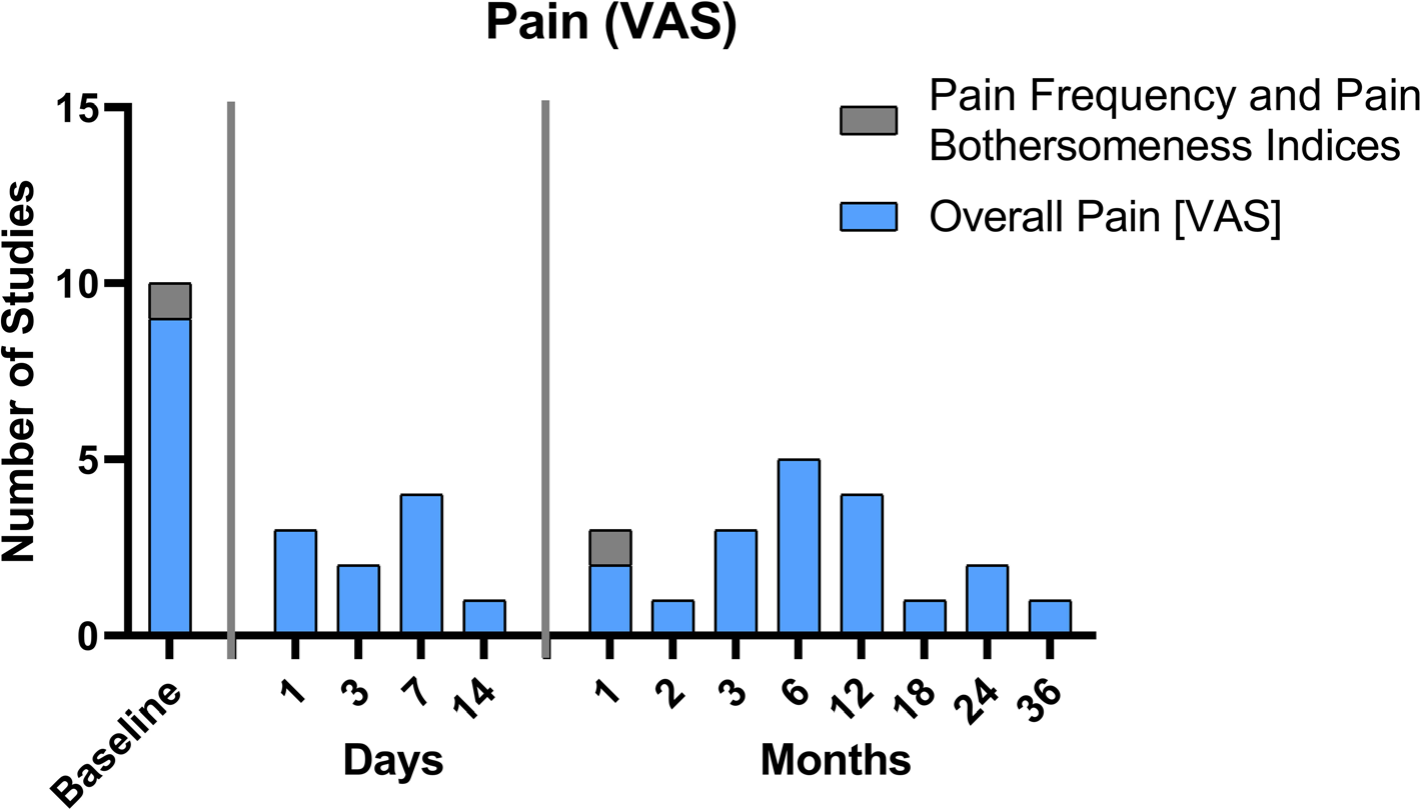
Overview of the time points at which pain assessment was collected using the Visual Analogue Scale (VAS) in the nine studies analyzed

A *Visual or Numeric Rating Scale scale (VAS and NRS respectively)* is an easy and widely used instrument for pain measurement [24, 25]. Five of the nine studies defined pain as their primary outcome (Table 2). Furthermore, the pain was measured in every study at least at baseline. In the short term, four of nine studies assessed pain after 1 week. The most frequently used long term time points were 3, 6, and 12 months (Fig. 2, Table S1 additional files).

Other measures of pain were the *Pain Frequency and Pain Bothersomeness Indices* each measured on a 0 to 4-point scale, with higher scores indicating more severe pain [11]. This questionnaire was used by only one study at baseline and one month follow up, making it the least frequently used questionnaire (Fig. 4, Table S1 additional file).

### Health-related quality of life (HRQoL)

Numerous questionnaires are available for recording HRQoL. In the nine studies analyzed, a total of five different instruments were used.

**Fig. 3:**
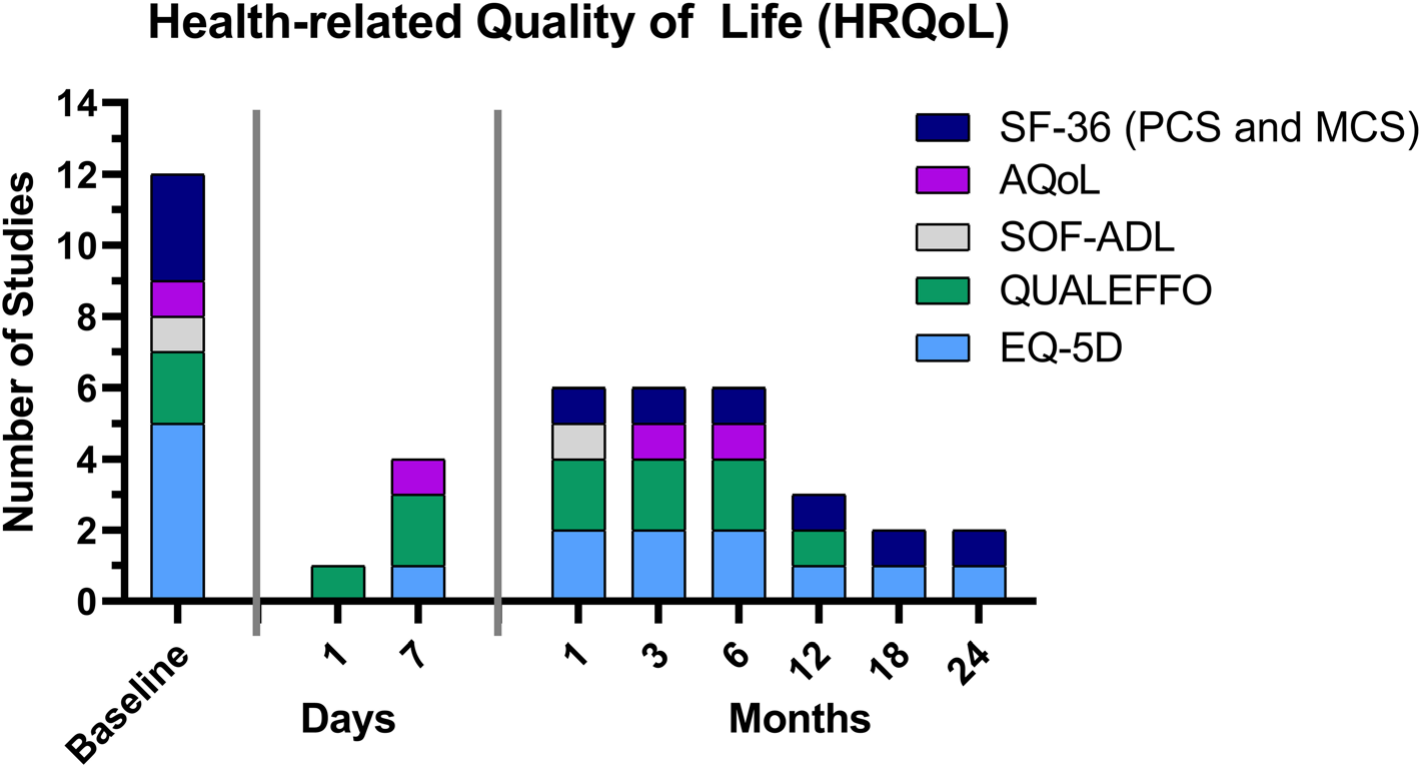
Overview of the time points at which the different questionnaires from the health-related quality of life (HRQoL) category were evaluated in the nine studies analyzed. QUALEFO = Questionnaire of the European Foundation for Osteoporosis; AqoL = The Assessment of Quality of Life; EQ-5D = European Quality of Life–5 Dimensions; SF-36 = Short Form 36 General Health Survey (MCS = mental component score, PCS = physical component score); SOF-ADL = Study of Osteoporotic Fractures-Activities of Daily Living

The *European Quality of Life–5 Dimensions (EQ–5D)* scale (scale from 0 to 1, where 1 indicates perfect health) is a commonly used questionnaire that is also free of charge [26–28]. Five of the nine studies collected the EQ-5D at baseline, while four studies had also collected follow-up data (Fig. 3, Table S1 additional file).

The *Short Form 36 General Health Survey (SF 36)* [29, 30] is also a well-known and commonly used measure to assess HRQoL. It averages the items of each subscale to generate a score ranging from 0 to 100, with a lower score representing greater disability [31]. In addition, the SF-36 has a physical and mental component score (PCS and MCS, respectively). Overall, the SF-36 was obtained at baseline and follow-up in three of the nine RCTs, but at different time points. (Fig. 3, Table S1 additional file).

The *Questionnaire of the European Foundation for Osteoporosis (QUALEFFO)* is a 41-item questionnaire specifically related to vertebral fractures and osteoporosis (scores range from 0 to 100, with lower scores indicating better quality of life) [32]. This questionnaire was used in two clinical trials [17, 18] (Fig. 3, Table S1 additional file).

There were additionally two other questionnaires used to measure HRQoL. One is the *Assessment of Quality of Life (AQoL)* questionnaire, which is a well-validated instrument sensitive to changes in the elderly and frail (scores range from 0 to 1, with 1 indicating perfect health) [33]. The other one was the *Study of Osteoporotic Fractures-Activities of Daily Living (SOF-ADL)* questionnaire, an easily obtained index to assess frailty [34]. However, these two questionnaires were only collected by one study, and the SOF-ADL was only collected once at baseline (Fig. 3, Table S1 additional file).

### Disability and function

Four different instruments were used to assess disability and function in the nine studies analyzed.

**Fig. 4:**
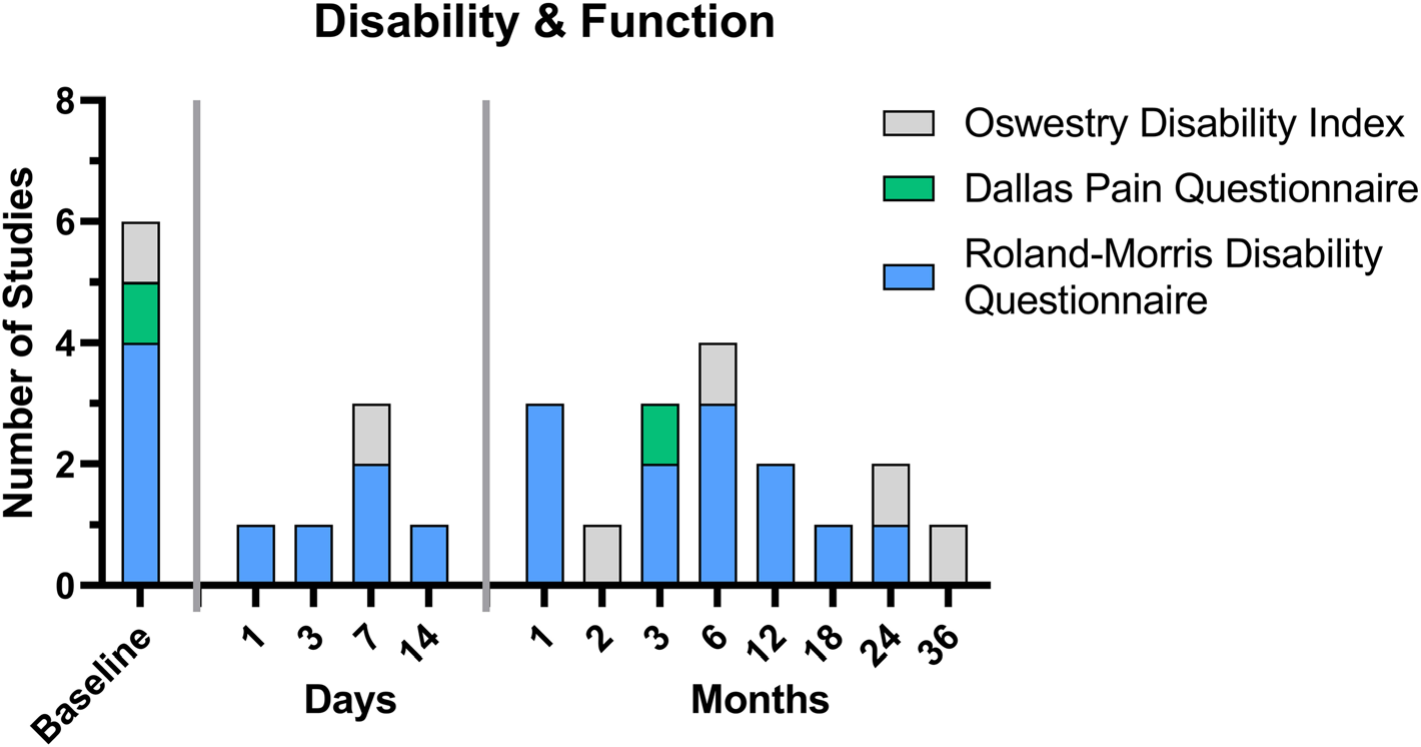
Overview of the time points at which the different questionnaires from the Disability and Function category were evaluated in the nine studies analyzed

The *Roland-Morris Disability Questionnaire (RMDQ)* is a widely used measure to assess health status in low back pain. It is designed to assess only physical disability due to low back pain [35] (scores range from 0 to 23, with higher numbers indicating worse physical function). Originally, the scale assessed 12 categories with 24 items [36], with the modified version including 23 items covering domains of daily living [31]. The RMDQ was used as a baseline measure by almost half of the RCTs analyzed (four of nine studies). Regarding follow-up measurements, the time points ranged from one day to two months. Three studies chose the same time points, after one and six months, for follow-up (Fig. 4, Table S1 additional file).

*The Oswestry Disability Index (ODI)* was developed in 1976 in a specialized referral clinic with a large number of patients with chronic low back pain [37]. This scale is a functional measure of HrQOL, which includes six items in 10 dimensions [38]. However, the ODI was only collected in one of nine studies [22] (Fig. 4, Table S1 additional file).

The *Dallas Pain Questionnaire (DPQ)* measures four categories (16-items) of impairment of daily living due to chronic low back pain (0% is no pain and 100% is constant pain) [31, 39]. The DPQ was used in only one study and at baseline and three-month follow-up [20] (Fig. 4, Table S1 additional file).

### Radiographic imaging

**Fig. 5:**
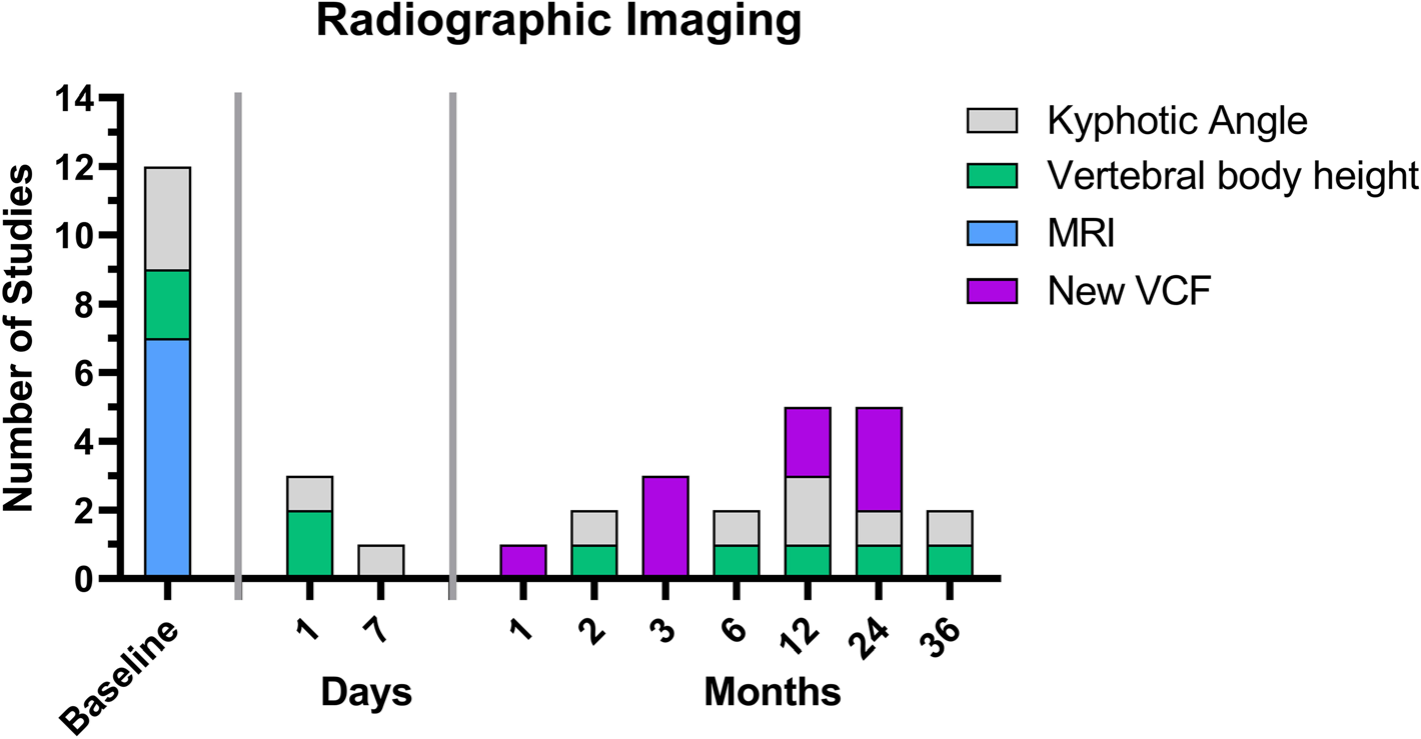
Overview of the time points at which radiographic follow up was evaluated in the nine studies analyzed. VCF Vertebral compression fracture, MRI = Magnetic Resonance Imaging

In all studies, VCFs were confirmed by radiological imaging. However, only seven of the nine studies analyzed performed initial imaging by spinal MRI. All follow-up examinations were performed using conventional radiographs. The most common outcome described was the occurrence of a new VCF (six of nine RCTs) and the kyphotic angle above the VCF (two of nine RCTs). Also, vertebral body height was measured and reported in three of the nine studies. However, the time points varied between studies. New VCFs were most frequently reported at three and 24 months, whereas kyphotic angle was most frequently measured at 12 months (Fig. 5, Table S1 additional file).

### Others

**Fig. 6:**
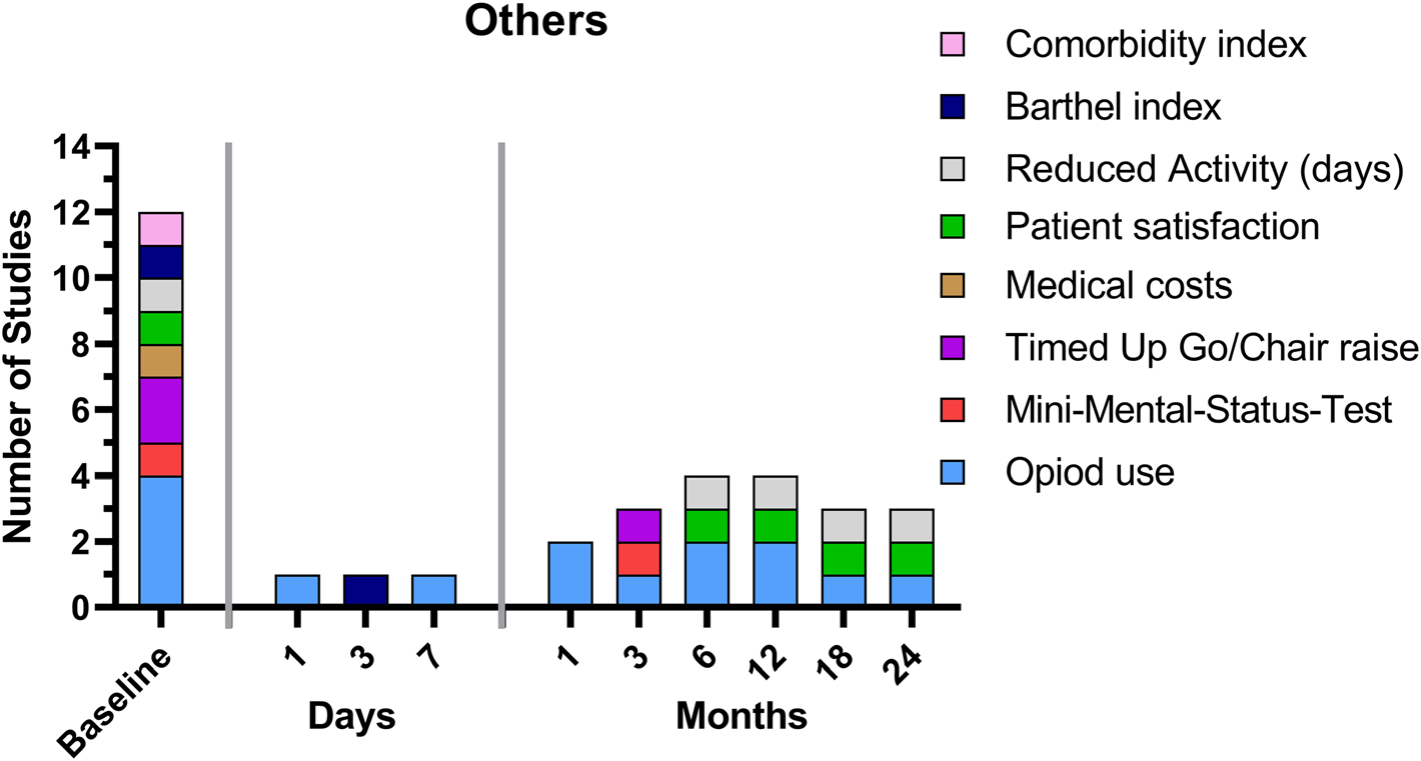
Overview of the various other outcome parameters and the times at which they were evaluated in the nine studies analyzed

In addition, other outcome measures were used in the highly cited RCTs analyzed. In addition to the patient-reported outcomes described above, opioid use was the most commonly described outcome parameter (four of nine studies) (Fig. 6, Table S1 additional file).

## Discussion

To the best of our knowledge, this is the first study to analyze the outcome measurements of frequently cited Level I studies on VCF treatment. A detailed analysis of the nine most frequently cited RCTs showed that a variety of questionnaires were used. In addition, there was a large difference between the survey dates of the questionnaires. Overall, in all included RCTs, pain (using VAS-pain) was the most common described outcome parameter, followed by HrQoL (using EQ-5D) and disability and function (using RMDQ). Objective outcome parameters (radiological imaging), however, were described less.

Due to the inconsistency in the reported (primary) outcome parameters of clinical studies, the comparability of the results and thus also the reliability of meta-analysis and systematic reviews are made more difficult. To conduct a high-quality clinical trial, like a level-I RCT, it is crucial to define and also declare specific outcome parameters. Moreover, one primary endpoint of a study should be determined, as it was already recommended in the 1996s’ CONsolidated Standards of Reporting Trials (CONSORT) statement to improve the reporting of randomised controlled trials [40]. Nevertheless, in a cohort of 519 published RCTs in 2000, fewer than half of the studies reported the primary outcome [41]. In the nine studies analyzed here, the primary outcome was reported in six of the nine RCTs.

The most often used outcome parameter in the studies analyzed was pain assessment by a VAS (or NRS). In this regard, our results are consistent with a recent systematic review of 401 included studies. The authors noted that the use of the VAS for pain intensity was not only the most commonly used outcome measure but also has increased in importance over the past three decades [42]. Overall, however, it should be kept in mind that while the VAS pain is an easy-to-use tool for pain assessment, this is also a highly subjective parameter that may not specifically indicate back pain.

In the HrQoL category, the most frequently collected measure was the EQ-5D [28]. This questionnaire is the most commonly used generic preference-based measure in clinical trials [43] and has also been cited by other authors as a commonly used outcome measure in low back pain studies [42]. Besides, the EQ-5D is part of the Standard Set Low back pain recommended by the International Consortium for Health Outcomes Measurement (ICHOM) [44]. However, a drawback could be that neither the EQ-5D nor the SF-36 are specific for low back pain in general and osteoporotic fracture pain in particular. Thus, a major criticism levelled against the use of generic HRQoL questionnaires is that these were designed to measure the quality of life across a wide range of conditions. Therefore, they may not be sensitive enough to measure a specific difference related to the disease of interest. Regarding our results, the QUALEFFO is the only vertebral fracture-specific outcome measure. It was used by only two of the RCTs analyzed [17, 18]. The other two questionnaires, SOF-ADL and AqOL, were not specifically designed for outcomes related to VCF but were at least developed for the elderly (geriatric) population [33, 34].

Disability and function were measured by most of the studies reviewed. Moreover, the third most commonly used outcome parameter overall was the RMDQ, which was used by four of the nine RCTs reviewed. The RMDQ has previously been shown to be an easy-to-administer, well-validated, back pain-specific outcome measure [45]. Although the ODI has been recommended as a back pain-specific measure of disability by researchers in this field [46], it was only used by the RCT conducted by Farrokhi et al. in 2011 [22]. The RMDQ, however, increasingly used in the early 2000s, lost popularity by 2012 [42].

Although measurement of various parameters using radiographic imaging has the advantage of being an objective parameter, it does not necessarily correlate with the patient’s condition. Despite the fact that there are several methods of measurement [47], radiological outcome measures focus mainly on kyphosis angle, reduction loss, and vertebral body height loss [48]. This is also consistent with the results of the RCTs that have been analyzed. Kyphosis angle and vertebral body height were the most frequently measured parameters. However, only slightly more than half of the studies reviewed also described radiological outcome parameters. Especially in VCFs, it is important to note that the detection of adjacent fractures influences the treatment of the underlying osteoporotic disease. To prevent severe osteoporotic spinal deformities [49], regular radiological follow-up should be essential, not only from the surgeon’s point of view. Particularly little is known about the mid-to-long-term follow-up of patients who have undergone vertebral cement augmentation techniques, and despite our findings, the question of new and adjacent fractures in particular, as well as the drug treatment of osteoporosis itself, needs to become a greater focus of level 1 studies.

For the sake of completeness, all “other” outcome parameters used should be mentioned here, of which the use of opioids was the most frequently evaluated. This is a good objective outcome parameter, although it is equally based on the subjective perception of pain.

Regarding the planning of follow-up examinations, to the best of our knowledge, no clear recommendations exist. In general, the scheduling of included follow-up examinations should have a pragmatic approach [50], e.g., following the standard follow-up protocol of the clinic. In the studies reviewed here, 1 week, 1, 6, 12, and 24 months were the most commonly set time points for follow-up.

Our study has limitations. Only nine RCTs met our inclusion criteria, biasing the results of our study [51]. Nonetheless, the included studies were high-quality level I studies that also had a high number of citations. Another limitation is, that we only used one database: The ‘Web of Science Core Collection’. W decided to use this database because it has been in existence since 1997 and is the world’s leading citation database. Moreover, it has been shown that for health sciences and medicine, the overlap of citations between Web of Science, Scopus and Google Schoolar is 91–95% [52]. Overall, we think that this extra effort would not outweigh the benefit. In addition, there is a possibility that some key outcome parameters may be overestimated, while others may be missing. Overall, the objective of this study, however, was to provide guidance for the design of future clinical trials and therefore focused on only a few, but highly influential, articles. All studies analyzed were published between 2009 and 2011, so age does not translate into higher citation density. As Aksnes et al. showed, even for highly cited articles, there is a decrease in citations starting five years after publication [53]. The citation rate is probably influenced by the number of authors involved and the breadth of the research field [53–55]. Methodological consistency seems to ensure a high citation rate [56]. Another limitation is that we cannot say whether or not there is an influence of the selected outcome parameters on the study result. However, the primary aim of this study was not to perform a meta-analysis but to reflect the conclusions of highly cited influential studies, which deal with the treatment of VCFs as this has already been done by Buchbinder [11] and Anderson et al. [57]. Because of the relationship between the research question and citation density, the ISI Web of Knowledge database was used exclusively because it is the only source for obtaining accurate citation information. Although we conducted a comprehensive data collection, we cannot exclude the possibility of missing articles, so this is another limitation of our study. The results of our study should help clinicians and researchers select appropriate outcome measures to conduct high-quality, comparable studies on the treatment of VCFs. As a consequence of our systematic literature review, it can be recommended to focus on the following outcome parameters when planning future clinical trials: EQ-5D (included the VAS pain), RMDQ, opioid use and a radiographic outcome Nevertheless, further research must address the question of HRQoL scores are sufficient to adequately address the outcome of interventional procedures in traumatic fracture situations.

## Conclusion

With our study, we demonstrated that a large inconsistency exists between outcome measures in highly cited Level I studies of VCF treatment. Pain (VAS), followed by HrQoL (EQ-5D) and disability and function (RMDQ), opioid use, and radiological outcome (kyphotic angle, VBH, and new VCFs) were the most commonly used outcome parameters and should be considered when defining the outcome parameters of a study. Consensus on outcome parameters may improve the relevance of a study and for further comparisons in meta-analyses.

## Supplementary Information

**Additional file 1: Table S1.** Detailed overview of the included studies. VAS = Visual analogue scale, QUALEFFO = Questionnaire of the European Foundation for Osteoporosis; AQoL=The Assessment of Quality of Life, EQ-5D = European Quality of Life–5 Dimensions, SF 36 = Short Form 36 = General Health Survey, MCS = Mental component score, PCS = Physical component score, ODI = Oswestry Disability Index, RMDQ = Roland–Morris Disability Questionnaire, DPQ = Dallas Pain Questionnaire; MRI = Magnetic Resonance Imaging; mmSE = Mini-Mental State Examination, VBH = Vertebral body height; KA = Kyphotic Angel, SI = Sagittal index; VCF = Vertebral compression fracture; CMI = Comorbidity Index † Overall pain, and pain at rest and in bed at night.

## Data Availability

The datasets generated and/or analyzed during the current study are available from the corresponding author on reasonable request.

## Abbreviations

AQoL: The Assessment of Quality of Life
CaP: Calcium phosphate,
CEBM: Oxford Centre for Evidence-Based Medicine
CONSORT: CONsolidated Standards of Reporting Trials
DALY: Disability-adjusted life years
DPQ: Dallas Pain Questionnaire
EQ-5D: European Quality of Life–5 Dimensions
HRQoL: Health-related quality of life
JBMR: Journal of Bone and Mineral Research
KP: Kyphoplasty
LBP: Low-back pain
MCS: Mental component score
MRI: Magnetic Resonance Imaging
NEJM: New England Journal of Medicine
NRS: Numeric Rating Scale
ODI: Oswestry Disability Index
PCS: Physical component score
PMMA: Polymethylmethacrylate
PRISMA: Preferred Reporting Items for Systematic Reviews and Meta-Analysis
PV: Percutaneous vertebroplasty
QOL: Quality of life
QUALEFFO: Questionnaire of the European Foundation for Osteoporosis
RCT: Randomized controlled trial
RMDQ: Roland–Morris Disability Questionnaire
SBI: Sciatica Bothersomeness Index
SF 36: Short Form 36 General Health Survey
SI: Sagittal index
SOF-ADL: Study of Osteoporotic Fractures-Activities of Daily Living
VAS: Visual analog scale
VB: Vertebral body
VBH: VB height
VCF: Vertebral compression fracture
VP: Vertebroplasty

## References

[1] Consensus development conference: Diagnosis, prophylaxis, and treatment of osteoporosis. In: The American Journal of Medicine. 1993. p. 646–50. 10.1016/0002-9343(93)90218-e. 10.1016/0002-9343(93)90218-e 8506892

[2] Hernlund E, Svedbom A, Ivergård M, Compston J, Cooper C, Stenmark J (2013). Osteoporosis in the European Union: medical management, epidemiology and economic burden. A report prepared in collaboration with the International Osteoporosis Foundation (IOF) and the European Federation of Pharmaceutical Industry Associations (EFPIA). Arch Osteoporos.

[3] Melton LJ, Chrischilles EA, Cooper C, Lane AW, Riggs BL (1992). Perspective how many women have osteoporosis?. J Bone Miner Res.

[4] Foundation IO. Facts and statistics | international Osteoporosis Foundation. Int Osteoporotic Found. 2017; https://www.iofbonehealth.org/facts-statistics. .

[5] Johnell O, Kanis JA. An estimate of the worldwide prevalence and disability associated with osteoporotic fractures. Osteoporos Int. 2006;17:1726–33. 10.1007/s00198-006-0172-4 16983459

[6] Jalava T, Sarna S, Pylkkänen L, Mawer B, Kanis JA, Selby P, et al. Association Between Vertebral Fracture and Increased Mortality in Osteoporotic Patients. J Bone Miner Res. 2003;18:1254–60. 10.1359/jbmr.2003.18.7.1254. 10.1359/jbmr.2003.18.7.1254 12854835

[7] Parreira PCS, Maher CG, Megale RZ, March L, Ferreira ML (2017). An overview of clinical guidelines for the management of vertebral compression fracture: a systematic review. Spine J.

[8] Lee JH, Lee J-H, Jin Y (2017). Surgical techniques and clinical evidence of vertebroplasty and kyphoplasty for osteoporotic vertebral fractures. Osteoporos Sarcopenia.

[9] Iolascon G, Moretti A, Toro G, Gimigliano F, Liguori S, Paoletta M (2020). Pharmacological therapy of osteoporosis: What’s new?. Clin Interv Aging.

[10] Kado DM, Browner WS, Palermo L, Nevitt MC, Genant HK, Cummings SR (1999). Vertebral fractures and mortality in older women: a prospective study. Arch Intern Med.

[11] Buchbinder R, Golmohammadi K, Johnston RV, Owen RJ, Homik J, Jones A, et al. Percutaneous vertebroplasty for osteoporotic vertebral compression fracture. Cochrane Database Syst Rev. 2015;2015. 10.1002/14651858.CD006349.pub2. 10.1002/14651858.CD006349.pub2 25923524

[12] Grimes DA, Schulz KF (2002). An overview of clinical research: the lay of the land. Lancet..

[13] Moher D, Liberati A, Tetzlaff J, Altman DG (2009). Preferred reporting items for systematic reviews and meta-analyses: the PRISMA statement. J Clin Epidemiol.

[14] Phillips B, Ball C, Sackett D, Badenoch D, Straus S, Haynes B, et al. Oxford Centre for Evidence-based Medicine - levels of evidence (March 2009) - CEBM. 1998;1998(8):5515–20. 10.1073/pnas.082117599.

[15] Boonen S, Van Meirhaeghe J, Bastian L, Cummings SR, Ranstam J, Tillman JB (2011). Balloon kyphoplasty for the treatment of acute vertebral compression fractures: 2-year results from a randomized trial. J Bone Miner Res.

[16] Kallmes DF, Comstock BA, Heagerty PJ, Turner JA, Wilson DJ, Diamond TH, Edwards R, Gray LA, Stout L, Owen S, Hollingworth W, Ghdoke B, Annesley-Williams DJ, Ralston SH, Jarvik JG (2009). A randomized trial of vertebroplasty for osteoporotic spinal fractures. N Engl J Med.

[17] Buchbinder R, Osborne RH, Ebeling PR, Wark JD, Mitchell P, Wriedt C, Graves S, Staples MP, Murphy B (2009). A randomized trial of vertebroplasty for painful osteoporotic vertebral fractures. N Engl J Med.

[18] Klazen CAH, Lohle PNM, De Vries J, Jansen FH, Tielbeek AV, Blonk MC (2010). Vertebroplasty versus conservative treatment in acute osteoporotic vertebral compression fractures (Vertos II): an open-label randomised trial. Lancet..

[19] Klazen CAH, Venmans A, De Vries J, Van Rooij WJ, Jansen FH, Blonk MC (2010). Percutaneous vertebroplasty is not a risk factor for new osteoporotic compression fractures: results from VERTOS II. Am J Neuroradiol.

[20] Rousing R, Hansen KL, Andersen MO, Jespersen SM, Thomsen K, Lauritsen JM (2010). Twelve-months follow-up in forty-nine patients with acute/semiacute osteoporotic vertebral fractures treated conservatively or with percutaneous vertebroplasty: A clinical randomized study. Spine (Phila Pa 1976).

[21] Liu JT, Liao WJ, Tan WC, Lee JK, Liu CH, Chen YH, Lin TB (2010). Balloon kyphoplasty versus vertebroplasty for treatment of osteoporotic vertebral compression fracture: a prospective, comparative, and randomized clinical study. Osteoporos Int.

[22] Farrokhi MR, Alibai E, Maghami Z (2011). Randomized controlled trial of percutaneous vertebroplasty versus optimal medical management for the relief of pain and disability in acute osteoporotic vertebral compression fractures: clinical article. J Neurosurg Spine.

[23] Blattert TR, Jestaedt L, Weckbach A (2009). Suitability of a calcium phosphate cement in osteoporotic vertebral body fracture augmentation. Spine (Phila Pa 1976).

[24] Farrar JT, Young JP, LaMoreaux L, Werth JL, Poole MR (2001). Clinical importance of changes in chronic pain intensity measured on an 11-point numerical pain rating scale. Pain..

[25] Huskisson EC (1974). Measurement of pain. The Lancet.

[26] Walters SJ, Brazier JE (2005). Comparison of the minimally important difference for two health state utility measures: EQ-5D and SF-6D. Qual Life Res.

[27] Rabin R, de Charro F (2001). EQ-5D: a measure of health status from the EuroQol group. Ann Med.

[28] EuroQol Group (1990). EuroQol - a new facility for the measurement of health-related quality of life. Health Policy (New York).

[29] Ware JE, Kosinski MA, Keller SD (1994). SF-36 Physical and Mental Health Summary Scales.

[30] Laucis NC, Hays RD, Bhattacharyya T (2014). Scoring the SF-36 in orthopaedics: a brief guide. J Bone Jt Surg - Am Vol.

[31] Chapman JR, Hanson BP, Dettori JR, Norvell DC (2014). Spine outcomes measures and instruments.

[32] Lips P, Cooper C, Agnusdei D, Caulin F, Egger P, Johnell O, Kanis JA, Kellingray S, Leplege A, Liberman UA, McCloskey E, Minne H, Reeve J, Reginster JY, Scholz M, Todd C, de Vernejoul MC, Wiklund I (1999). Quality of life in patients with vertebral fractures: validation of the quality of life questionnaire of the European Foundation for Osteoporosis (QUALEFFO). Osteoporos Int.

[33] Hawthorne G, Osborne R (2005). Population norms and meaningful differences for the assessment of quality of life (AQoL) measure. Aust N Z J Public Health.

[34] Ettinger B, Black DM, Nevitt MC, Rundle AC, Cauley JA, Cummings SR, Genant HK (1992). Contribution of vertebral deformities to chronic back pain and disability. J Bone Miner Res.

[35] Roland M, Fairbank J (2000). The Roland-Morris disability questionnaire and the Oswestry disability questionnaire. Spine (Phila Pa 1976).

[36] ROLAND M, MORRIS R (1983). A study of the natural history of back pain. Spine (Phila Pa 1976).

[37] Müller U, Duetz MS, Roeder C, Greenough CG (2004). Condition-specific outcome measures for low back pain: part I: validation. Eur Spine J.

[38] Fairbank JCT, Davies JB, Couper J, O’Brien JP (1980). The Oswestry low back pain disability questionnaire. Physiotherapy..

[39] Lawlis GF, Cuencas R, Selby D, McCoy CE (1989). The development of the dallas pain questionnaire an assessment of the impact of spinal pain on behavior. Spine (Phila Pa 1976).

[40] Begg C, Cho M, Eastwood S, Horton R, Moher D, Olkin I, Pitkin R, Rennie D, Schulz KF, Simel D, Stroup DF (1996). Improving the quality of reporting of randomized controlled trials: the CONSORT statement. J Am Med Assoc.

[41] Consort-Statement > CONSORT 2010 > Outcomes. http://www.consort-statement.org/checklists/view/32%2D%2Dconsort-2010/80-outcomes. Accessed 3 January 2021.

[42] Froud R, Patel S, Rajendran D, Bright P, Bjørkli T, Buchbinder R, et al. A systematic review of outcome measures use, analytical approaches, reporting methods, and publication volume by year in low back pain trials published between 1980 and 2012: Respiece, adspice, et prospice. PLoS One. 2016(10):11. 10.1371/journal.pone.0164573. 10.1371/journal.pone.0164573 PMC5077121 27776141

[43] Payakachat N, Ali MM, Tilford JM (2015). Can the EQ-5D detect meaningful change? a systematic review. Pharmacoeconomics.

[44] Low Back Pain – ICHOM Connect. https://connect.ichom.org/standard-sets/low-back-pain/. Accessed 1 April 2021.

[45] Trout AT, Kallmes DF, Gray LA, Goodnature BA, Everson SL, Comstock BA, Jarvik JG (2005). Evaluation of vertebroplasty with a validated outcome measure: the Roland-Morris disability questionnaire. Am J Neuroradiol.

[46] Smeets R, Köke A, Lin CW, Ferreira M, Demoulin C (2011). Measures of function in low back pain/disorders: Low Back Pain Rating Scale (LBPRS), Oswestry Disability Index (ODI), Progressive Isoinertial Lifting Evaluation (PILE), Quebec Back Pain Disability Scale (QBPDS), and Roland-Morris Disability Questionnaire. Arthritis Care Res.

[47] Hsu WE, Su KC, Chen KH, Pan CC, Lu WH, Lee CH (2019). The evaluation of different radiological measurement parameters of the degree of collapse of the vertebral body in vertebral compression fractures. Appl Bionics Biomech.

[48] Schoenfeld AJ, Bono CM (2011). Measuring spine fracture outcomes: common scales and checklists. Injury..

[49] Yuan HA, Brown CW, Phillips FM (2004). Osteoporotic spinal deformity: a biomechanical rationale for the clinical consequences and treatment of vertebral body compression fractures. J Spinal Disord Tech.

[50] Kaur M, Sprague S, Ignacy T, Thoma A, Bhandari M, Farrokhyar F. How to optimize participant retention and complete follow-up in surgical research. Can J Surg. 2014;57(6):420–7. 10.1503/cjs.006314. 10.1503/cjs.006314 PMC4245274 25421086

[51] MacRoberts MH, MacRoberts BR. Problems of citation analysis: a critical review. J Am Soc Inf Sci. 1989;40(5):342–9. 10.1002/(SICI)1097-4571(198909)40:5<342::AID-ASI7>3.0.CO;2-U.

[52] Martín-Martín A, Orduna-Malea E, Thelwall M, López-Cózar ED (2018). Google scholar, web of science, and Scopus: a systematic comparison of citations in 252 subject categories. J Inf Secur.

[53] Aksnes DW. Characteristics of highly cited papers. Res Eval. 2003;12(3):159–70. 10.3152/147154403781776645.

[54] Tahamtan I, Safipour Afshar A, Ahamdzadeh K (2016). Factors affecting number of citations: a comprehensive review of the literature. Scientometrics..

[55] Waltman L (2016). A review of the literature on citation impact indicators. J Inf Secur.

[56] Ahmad SSS, Meyer JC, Krismer AM, Ahmad SSS, Evangelopoulos DS, Hoppe S (2017). Outcome measures in clinical ACL studies: an analysis of highly cited level I trials. Knee Surg Sport Traumatol Arthrosc.

[57] Anderson PA, Froyshteter AB, Tontz WL (2013). Meta-analysis of vertebral augmentation compared with conservative treatment for osteoporotic spinal fractures. J Bone Miner Res.

