# Supplementary material for: “How to measure the outcome in the surgical treatment of vertebral compression fractures? A systematic literature review of highly cited level-I studies”
Source: BMC Musculoskelet Disord. 2021 Jun 24;22:579. doi: 10.1186/s12891-021-04305-6 (PMC8223299; doi:10.1186/s12891-021-04305-6)
Supplement: Supplementary file 1 — Additional file 1: Table S1. Detailed overview of the included studies. VAS = Visual analogue scale, QUALEFFO = Questionnaire of the European Foundation for Osteoporosis; AQoL=The Assessment of Quality of Life, EQ-5D = European Quality of Life–5 Dimensions, SF 36 = Short Form 36 = General Health Survey, MCS = Mental component score, PCS = Physical component score, ODI = Oswestry Disability Index, RMDQ = Roland–Morris Disability Questionnaire, DPQ = Dallas Pain Questionnaire; MRI = Magnetic Resonance Imaging; mmSE = Mini-Mental State Examination, VBH = Vertebral body height; KA = Kyphotic Angel, SI = Sagittal index; VCF = Vertebral compression fracture; CMI = Comorbidity Index † Overall pain, and pain at rest and in bed at night. [file 12891_2021_4305_MOESM1_ESM.pptx]

## Slide 1
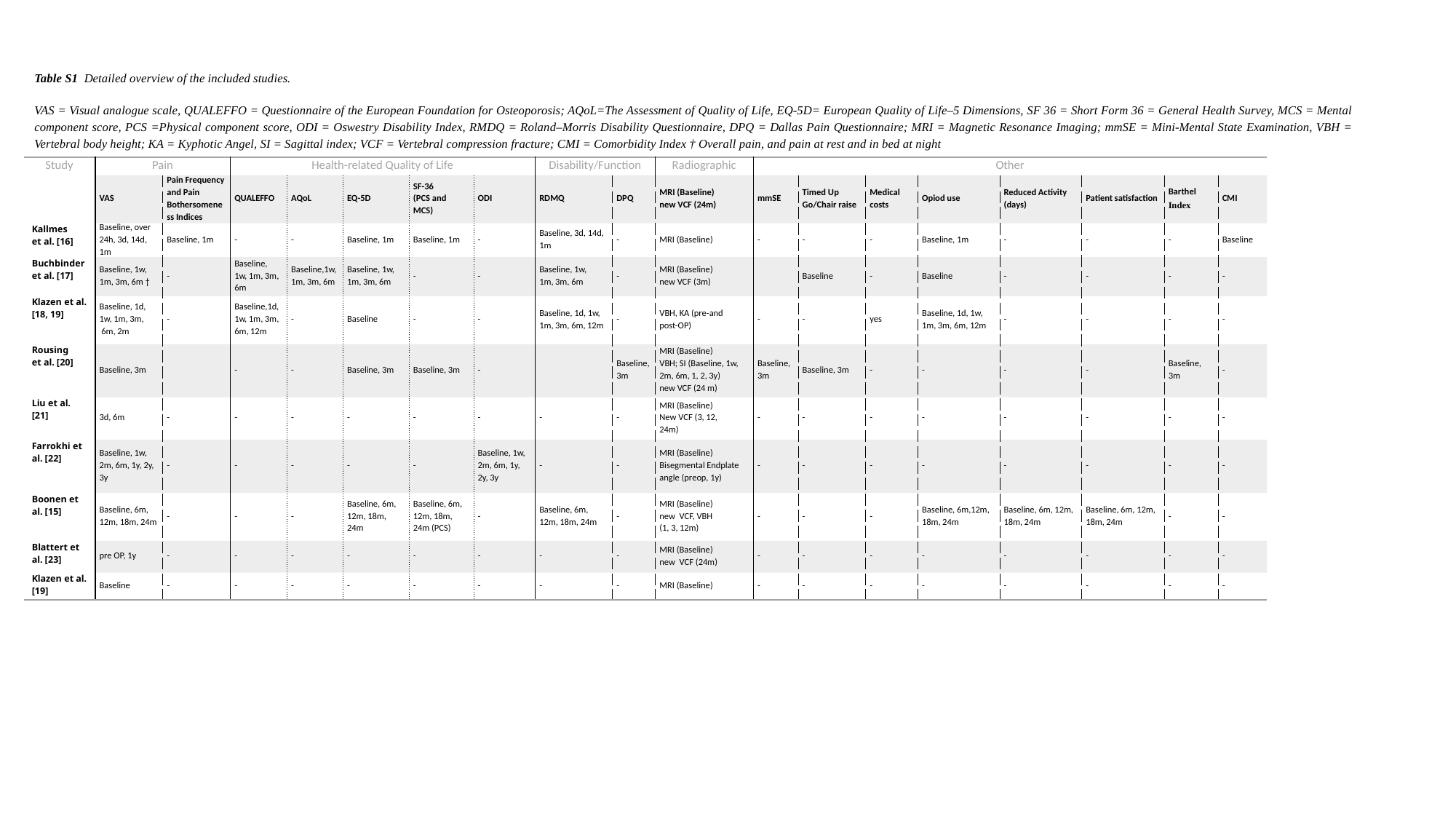

Table S1 Detailed overview of the included studies.
VAS = Visual analogue scale, QUALEFFO = Questionnaire of the European Foundation for Osteoporosis; AQoL=The Assessment of Quality of Life, EQ-5D= European Quality of Life–5 Dimensions, SF 36 = Short Form 36 = General Health Survey, MCS = Mental component score, PCS =Physical component score, ODI = Oswestry Disability Index, RMDQ = Roland–Morris Disability Questionnaire, DPQ = Dallas Pain Questionnaire; MRI = Magnetic Resonance Imaging; mmSE = Mini-Mental State Examination, VBH = Vertebral body height; KA = Kyphotic Angel, SI = Sagittal index; VCF = Vertebral compression fracture; CMI = Comorbidity Index † Overall pain, and pain at rest and in bed at night
| Study | Pain | | Health-related Quality of Life | | | | | Disability/Function | | Radiographic | Other | | | | | | | |
| --- | --- | --- | --- | --- | --- | --- | --- | --- | --- | --- | --- | --- | --- | --- | --- | --- | --- | --- |
| | VAS | Pain Frequency and Pain Bothersomene ss Indices | QUALEFFO | AQoL | EQ-5D | SF-36 (PCS and MCS) | ODI | RDMQ | DPQ | MRI (Baseline) new VCF (24m) | mmSE | Timed Up Go/Chair raise | Medical costs | Opiod use | Reduced Activity (days) | Patient satisfaction | Barthel Index | CMI |
| Kallmes et al. [16] | Baseline, over 24h, 3d, 14d, 1m | Baseline, 1m | - | - | Baseline, 1m | Baseline, 1m | - | Baseline, 3d, 14d, 1m | - | MRI (Baseline) | - | - | - | Baseline, 1m | - | - | - | Baseline |
| Buchbinder et al. [17] | Baseline, 1w, 1m, 3m, 6m † | - | Baseline, 1w, 1m, 3m, 6m | Baseline,1w, 1m, 3m, 6m | Baseline, 1w, 1m, 3m, 6m | - | - | Baseline, 1w, 1m, 3m, 6m | - | MRI (Baseline) new VCF (3m) | | Baseline | - | Baseline | - | - | - | - |
| Klazen et al. [18, 19] | Baseline, 1d, 1w, 1m, 3m, 6m, 2m | - | Baseline,1d, 1w, 1m, 3m, 6m, 12m | - | Baseline | - | - | Baseline, 1d, 1w, 1m, 3m, 6m, 12m | - | VBH, KA (pre-and post-OP) | - | - | yes | Baseline, 1d, 1w, 1m, 3m, 6m, 12m | - | - | - | - |
| Rousing et al. [20] | Baseline, 3m | | - | - | Baseline, 3m | Baseline, 3m | - | | Baseline, 3m | MRI (Baseline) VBH; SI (Baseline, 1w, 2m, 6m, 1, 2, 3y) new VCF (24 m) | Baseline, 3m | Baseline, 3m | - | - | - | - | Baseline, 3m | - |
| Liu et al. [21] | 3d, 6m | - | - | - | - | - | - | - | - | MRI (Baseline) New VCF (3, 12, 24m) | - | - | - | - | - | - | - | - |
| Farrokhi et al. [22] | Baseline, 1w, 2m, 6m, 1y, 2y, 3y | - | - | - | - | - | Baseline, 1w, 2m, 6m, 1y, 2y, 3y | - | - | MRI (Baseline) Bisegmental Endplate angle (preop, 1y) | - | - | - | - | - | - | - | - |
| Boonen et al. [15] | Baseline, 6m, 12m, 18m, 24m | - | - | - | Baseline, 6m, 12m, 18m, 24m | Baseline, 6m, 12m, 18m, 24m (PCS) | - | Baseline, 6m, 12m, 18m, 24m | - | MRI (Baseline) new VCF, VBH (1, 3, 12m) | - | - | - | Baseline, 6m,12m, 18m, 24m | Baseline, 6m, 12m, 18m, 24m | Baseline, 6m, 12m, 18m, 24m | - | - |
| Blattert et al. [23] | pre OP, 1y | - | - | - | - | - | - | - | - | MRI (Baseline) new VCF (24m) | - | - | - | - | - | - | - | - |
| Klazen et al. [19] | Baseline | - | - | - | - | - | - | - | - | MRI (Baseline) | - | - | - | - | - | - | - | - |
